# Trends in infant mortality and stillbirth rates in Scotland by socio-economic position, 2000–2018: a longitudinal ecological study

**DOI:** 10.1186/s12889-021-10928-0

**Published:** 2021-05-27

**Authors:** Alice Harpur, Jon Minton, Julie Ramsay, Gerry McCartney, Lynda Fenton, Harry Campbell, Rachael Wood

**Affiliations:** 1 The Usher Institute, The University of Edinburgh, Edinburgh, UK; 2 Department of Public Health NHS Lothian, Edinburgh, UK; 3 Public Health Scotland, Glasgow, UK; 4 National Records of Scotland, Edinburgh, UK

**Keywords:** Infant mortality, Stillbirth, Inequality

## Abstract

**Background:**

As Scotland strives to become a country where children flourish in their early years, it is faced with the challenge of socio-economic health inequalities, which are at risk of widening amidst austerity policies. The aim of this study was to explore trends in infant mortality rates (IMR) and stillbirth rates by socio-economic position (SEP) in Scotland, between 2000 and 2018, inclusive.

**Methods:**

Data for live births, infant deaths, and stillbirths between 2000 and 2018 were obtained from National Records of Scotland. Annual IMR and stillbirth rates were calculated and visualised for all of Scotland and when stratified by SEP. Negative binomial regression models were used to estimate the association between SEP and infant mortality and stillbirth events, and to assess for break points in trends over time. The slope (SII) and relative (RII) index of inequality compared absolute and relative socio-economic inequalities in IMR and stillbirth rates before and after 2010.

**Results:**

IMR fell from 5.7 to 3.2 deaths per 1000 live births between 2000 and 2018, with no change in trend identified. Stillbirth rates were relatively static between 2000 and 2008 but experienced accelerated reduction from 2009 onwards. When stratified by SEP, inequalities in IMR and stillbirth rates persisted throughout the study and were greatest amongst the sub-group of post-neonates. Although comparison of the SII and RII in IMR and stillbirths before and after 2010 suggested that inequalities remained stable, descriptive trends in mortality rates displayed a 3-year rise in the most deprived quintiles from 2016 onwards.

**Conclusion:**

Whilst Scotland has experienced downward trends in IMR and stillbirth rates between 2000 and 2018, the persistence of socio-economic inequalities and suggestion that mortality rates amongst the most deprived groups may be worsening warrants further action to improve maternal health and strengthen support for families with young children.

## Background

Due to improvements in sanitation and nutrition, adoption of family planning, and advancements in healthcare, the United Kingdom (UK) experienced large reductions in infant mortality rates (IMR) during the late 1800s and much of the 1900s [1, 2]. Since the 1990s, however, trends have been less encouraging. From 1990 onwards the UK’s downward IMR trajectory flattened and fell behind that of other high-income countries, with projections estimating that by 2030 the UK’s IMR could be 80% higher than the median rate of comparable countries [3]. More recently, an unusual 4-year consecutive rise in IMR in England was observed between 2014 and 2017 and whilst this trend was subsequently attributed to increasing numbers of early neonatal deaths of infants born at < 24^+ 0^ weeks gestation, improvements in IMR after exclusion of these events still appeared to be slowing, with annual decreases of 0.14 and 0.04 per 1000 live births before and after 2014, respectively. Persistent socio-economic inequalities in IMR in the UK are also a cause for concern, with infants from the most deprived areas in England having a 94% higher risk of death compared to infants from the least deprived areas [4, 5].

Although causal relationships have not been confirmed, the UK’s socio-economic environment has been cited as contributing to the recent trends in IMR and socio-economic inequalities. Following the 2008 global financial crisis the UK Government introduced an austerity programme in 2010, which has made significant changes to the UK’s tax and benefit systems, and to public spending. Concerns have been raised that these reforms have had a disproportionately negative impact upon the most deprived children in the UK [5]. For example, the two-child benefit limit which was introduced in 2017 has resulted in low-income families who have a third or subsequent child losing entitlement to additional support that equates to £2780 per child per year. Evaluating the impact of this policy, the Scottish Government predicted that 20,000 households would drop into relative poverty after housing costs [6]. The impact of austerity on mortality and socio-economic inequalities have also been postulated to extend beyond the early years, with studies hypothesising that austerity may be contributing to the observation of stalling and in some instances worsening life expectancy in high-income countries [7].

Looking beyond the UK, the impact of the 2008 global financial crisis and subsequent national fiscal policies on child health outcomes and socio-economic inequalities have also been explored elsewhere. On review of perinatal health outcomes, Greece observed adverse effects on low birth weight, preterm birth and infant mortality rates following the 2008 financial crisis and reported that those in long term unemployment and experiencing the greatest income reduction were at increased risk [8, 9]. Conversely, despite over 90% of its banking system collapsing in 2008, Iceland observed no significant change in maternal, perinatal, neonatal, infant or child mortality rates and cited governmental policies that protected vulnerable groups, including children and families, as alleviating negative health effects of the crisis [10].

As the impact that early life health can have on later life course outcomes and health inequalities is widely recognised [11, 12], it is vital that trends in child health outcomes are examined, using robust indicators such as IMR, so that policy makers are aware of potential impacts that social and fiscal policies have on early life health and that the need for interventions to improve early life health can be recognised promptly.

In Scotland, the most recent analysis of IMR by socio-economic position (SEP) was conducted between 1981 and 2011. This study showed downward trends in early neonatal, late neonatal, and post-neonatal mortality rates across all SEP groups but reported persistent socio-economic inequalities. During the study period absolute inequality remained stable amongst early neonates, increased for late-neonates, and declined amongst post-neonates [13]. However, as the study concluded in 2011, it could not capture the impact of austerity.

In the context of concerning trends elsewhere in the UK, and with a strong policy focus on early life health in Scotland, this study aimed to investigate trends in IMR by SEP in Scotland, between 2000 and 2018 and examine if there was a change in absolute or relative inequality before and after the introduction of austerity in 2010. As the most common causes of death vary by age at death, we also analysed trends in the sub-groups of neonatal mortality (infant deaths at 0–27 days of life) and post-neonatal mortality (infant deaths at 28 days to < 1 year of life). Additionally, as stillbirths, which are defined in the UK as infants delivered at or beyond 24^+ 0^ weeks gestation who do not breathe or show any other signs of life, are closely related to and can enhance the understanding of trends in IMR, we incorporated these into analysis using the extended perinatal mortality rate, which measures neonatal deaths and stillbirths per 1000 (live and still) births [14].

## Methods

### Protocol

The methodology described adhered to a study protocol that was written and published prior to commencing statistical analyses, available at: dx.doi.org/10.17504/protocols.io.bgxmjxk6. Deviations from the original protocol are noted in the relevant sections below.

### Data source and study design

This study adopted a longitudinal ecological study design set in Scotland. Datasets containing information about all live births, stillbirths, and infant deaths in Scotland between 2000 and 2018 were obtained from the National Records of Scotland (NRS).

### Exposures

The Scottish Index of Multiple Deprivation income and employment (SIMD-IE) score, which is a standard indicator of deprivation when performing health inequality analysis, and which avoids the potential of circular logic by removal of the health domain, was used as an area-level measure of SEP. SIMD-IE scores were collapsed into population-weighted quintiles and each event was assigned a quintile according to data zone at birth. The use of population-weighting adhered to guidance issued by Public Health Scotland; by accounting for variations in population sizes within data zones, population-weighting enables approximately the same percentage of the population to be captured in each quintile. The data zone and SIMD version appropriate to the year of each event was used [15].

**Fig. 1:**
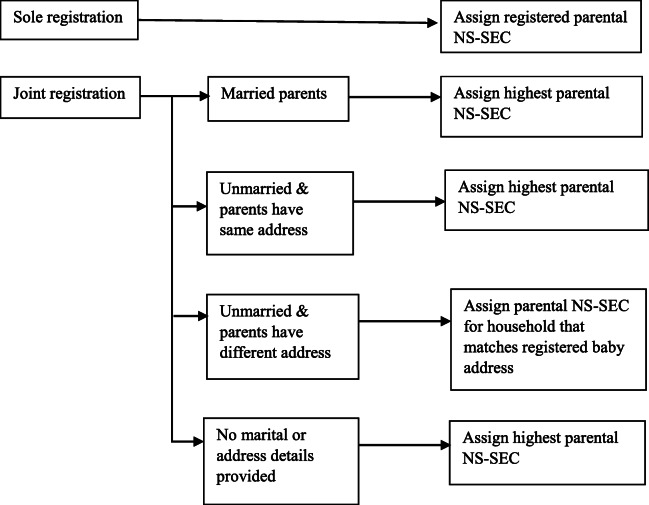
Flow diagram outlining allocation of highest-household parental NS-SEC

To overcome the risk of ecological fallacy and misclassification bias that arises from SIMD-IE, analyses were repeated using an individual-level measure of SEP based upon occupational social class. A modified version of the five-class National Statistics Socio-economic classification (NS-SEC) was assigned to each event using parental occupation at birth [16]. Details of parental marital status and living arrangements were used to assign a highest household NS-SEC by following the flowchart displayed in Fig. 1.

Adjustment for additional exposure variables when exploring the relationship between SEP and infant mortality and stillbirth was limited to those which are routinely collected during vital registration. These included infant sex and maternal age at birth, collapsed into three categories: < 25-years, 25–34-years and ≥ 35-years.

### Outcomes

The primary outcome of interest was IMR per 1000 live births, which was further analysed in subgroups according to age at death as neonatal mortality rate and post-neonatal mortality rate, per 1000 live births. The secondary outcomes of interest were stillbirth rates per 1000 (live and still) births, and the extended perinatal mortality rate per 1000 (live and still) births. The formulae used to calculate all mortality and stillbirth rates are presented in Table 1.

**Table 1:** Formulae used to calculate mortality and stillbirth event rates

Mortality event	Calculation
Infant mortality rate	$\frac{\mathrm{Deaths}\ \mathrm{aged}\ 0\;\mathrm{days}\ \mathrm{to}<1-\mathrm{year}}{\mathrm{Live}\ \mathrm{births}}\times 1000$
Neonatal mortality rate	$\frac{\mathrm{Deaths}\ \mathrm{aged}\ 0-27\;\mathrm{days}}{\mathrm{Live}\ \mathrm{births}}\times 1000$
Post-neonatal mortality rate	$\frac{\mathrm{Deaths}\ \mathrm{aged}\ 28\;\mathrm{days}\ \mathrm{to}<1-\mathrm{year}}{\mathrm{Live}\ \mathrm{births}}\times 1000$
Stillbirth rate	$\frac{\mathrm{Stillbirths}\;}{\mathrm{Live}\ \mathrm{births}+\mathrm{Stillbirths}}\times 1000$
Extended perinatal mortality rate	$\frac{\mathrm{Stillbirths}+\mathrm{Deathsaged}\kern0ex 0-27-\mathrm{days}}{\mathrm{Livebirths}+\mathrm{Stillbirths}}\times 1000$

### Statistical analyses

Infants with incomplete data on the exposure and/or confounding variables were excluded from analyses. Descriptive analyses began by calculating counts and proportions of live births, stillbirths and infant deaths by year, SIMD-IE, infant sex, and maternal age. Annual IMR per 1000 live births were calculated and trends over time were visualised.

To quantify the relationship between SEP and IMR, negative binomial regression models estimated the counts of infant mortality events by year and SIMD-IE, which were coded as numeric variables. The number of births in each year were included as an offset to account for annual fluctuations in birth rates and models were adjusted for infant sex, and maternal age. Decisions about which variables to include in the final model were guided by Bayesian Information Criterion (BIC) with smaller values indicating models that more efficiently balanced model complexity with model fit [17]. In the study protocol, adjustment for urban-rural classification was proposed but after obtaining the data this variable was found not to have a significant impact on the count of mortality or stillbirth events and adjustment for it did not improve the fit of the models, therefore a decision was made to exclude this variable from analyses.

To test for a change in trend in IMR over time, segmented regression analysis was performed. Annual break points were introduced sequentially to the negative binomial models, and BIC was used to assess which break point provided the best fitting model and if addition of a break point was superior to the absence of a break point.

To assess if socio-economic inequalities in IMR changed over time, the slope (SII) and relative (RII) indices of inequality were calculated using linear regression models. The calculated SII represented the absolute difference in IMR between the most and least deprived quintiles whilst the RII represented the SII as a proportion of the average IMR in the whole population, where a value of zero would indicate no inequality. To explore if changes in the inequality gap occurred following the introduction of the UK’s austerity programme in 2010, SII and RII were calculated and compared during two time periods: 2002–2010 (inclusive) and 2011–2018 (inclusive). An increase in the value of SII or RII post-austerity indicated worsening absolute and relative inequality, respectively.

Analyses were also conducted on the sub-groups of neonates, post-neonates, stillbirths and extended perinates, and were repeated using NS-SEC as an individual-level measure of SEP.

### Sensitivity analysis

As multiple pregnancies are associated with an increased risk of antenatal and intrapartum complications for both the mother and baby, which subsequently increases the risk of stillbirth and infant mortality, all analyses were repeated using infants from singleton pregnancies only.

## Results

### Study population

**Fig. 2:**
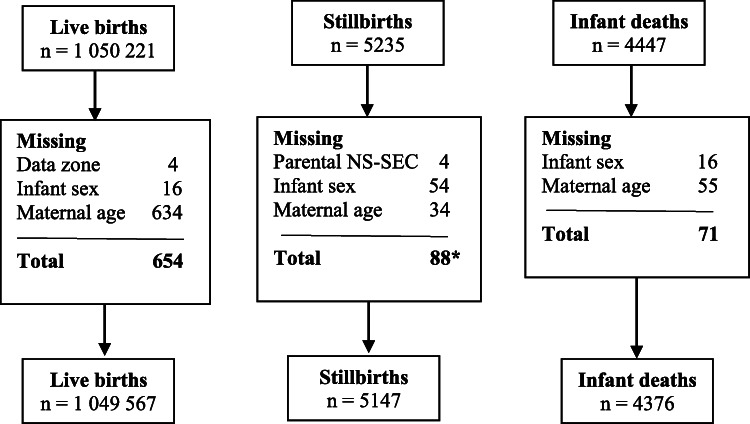
Overview of datasets included in analyses. Footnotes* 92 missing variables, which equated to 88 events as 4 events were missing both infant sex and maternal age

As displayed in Fig. 2, after exclusion of events with incomplete records, 1,049,567 live births, 5147 stillbirths and 4376 infant mortality events between 2000 and 2018 were included in analyses. As displayed in Table 2, stillbirth and mortality events were unequally distributed by SEP, with 29.3% of stillbirths and 32.0% of infant deaths occurring in the most deprived SIMD-IE quintile (Q1) compared to 14.4 and 13.2%, respectively, in the least deprived SIMD-IE quintile (Q5).

**Table 2:** Summary characteristics of live births, stillbirths, and infant deaths between 2000 and 2018: counts (%)

	Live births	Stillbirths	Neonatal deaths	Post-neonatal deaths	Total infant deaths	Extended perinatal deaths
**Total**						
	1,049,567 (100%)	5147 (100%)	2944 (100%)	1432 (100%)	4376 (100%)	8091 (100%)
**SIMD-IE Quintile**						
1 = Most deprived	257,252 (24.5%)	1510 (29.3%)	874 (29.7%)	526 (36.7%)	1400 (32.0%)	2384 (29.5%)
2	212,623 (20.3%)	1180 (22.9%)	681 (23.1%)	339 (23.7%)	1020 (23.3%)	1861 (23.0%)
3	196,925 (18.8%)	958 (18.6%)	501 (17.0%)	240 (16.8%)	741 (16.9%)	1459 (18.0%)
4	189,948 (18.1%)	757 (14.7%)	459 (15.6%)	180 (12.6%)	639 (14.6%)	1216 (15.0%)
5 = Least deprived	192,819 (18.4%)	742 (14.4%)	429 (14.6%)	147 (10.3%)	576 (13.2%)	1171 (14.5%)
**Parental Social Class**						
1 – Managerial &Professional	452,242 (43.1%)	1733 (33.7%)	1011 (34.3%)	390 (27.2%)	1401 (32.0%)	2744 (33.9%)
2 – Intermediate	168,567 (16.1%)	754 (14.6%)	442 (15.0%)	197 (13.8%)	639 (14.6%)	1196 (14.8%)
3 – Small employers	49,604 (4.7%)	236 (4.6%)	135 (4.6%)	76 (5.3%)	211 (4.8%)	371 (4.6%)
4 – Supervisors/craft related	61,692 (5.9%)	299 (5.8%)	177 (6.0%)	96 (6.7%)	273 (6.2%)	476 (5.9%)
5a – Semi-routine & routine	224,287 (21.4%)	1412 (27.4%)	768 (26.1%)	420 (29.3%)	1188 (27.1%)	2180 (26.9%)
5b – Other*	93,175 (8.9%)	713 (13.9%)	411 (14.0%)	253 (17.7%)	664 (15.2%)	1124 (13.9%)
**Infant Sex**						
Female	511,264 (48.7%)	2511 (48.8%)	1298 (44.1%)	615 (42.9%)	1913 (43.7%)	3809 (47.1%)
Male	538,303 (51.3%)	2636 (51.2%)	1646 (55.9%)	817 (57.1%)	2463 (56.3%)	4282 (52.9%)
**Maternal Age (years)**						
< 25	248,129 (23.6%)	1356 (26.3%)	825 (28.0%)	495 (34.6%)	1320 (30.2%)	2181 (27.0%)
25-34	591,972 (56.4%)	2596 (50.4%)	1519 (51.6%)	689 (48.1%)	2208 (50.5%)	4115 (50.9%)
35+	209,466 (20.0%)	1195 (23.2%)	600 (20.4%)	248 (17.3%)	848 (19.4%)	1795 (22.2%)
**Multiplicity status**						
Singleton birth	1,017,940 (97.0%)	4790 (93.1%)	2445 (83.1%)	1287 (89.9%)	3732 (85.3%)	8522 (89.5%)
Multiple birth	31,627 (3.0%)	357 (6.9%)	499 (16.9%)	145 (10.1%)	644 (14.7%)	1001 (10.5%)

* Never worked/long term unemployed, students and uncoded occupations

### Time trends in infant mortality and stillbirth rates over time

**Fig. 3:**
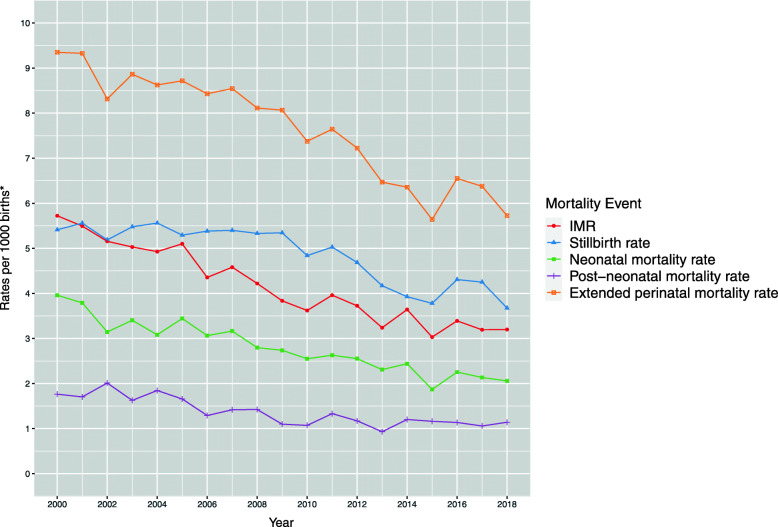
Trends in infant mortality and stillbirth rates, 2000–2018. Legend: * per 1000 live births for IMR, neonatal and post-neonatal mortality rates. Per 1000 live & stillbirths for stillbirth and extended perinatal mortality rates

Observing Fig. 3, between 2000 and 2018, the IMR in Scotland decreased from 5.7 to 3.2 deaths per 1000 live births. In the latter years the downward trends appeared to flatten, with IMR fluctuating between 3.2–3.3 deaths per 1000 live births between 2016 and 2018 but addition of break points to the negative binomial regression models did not improve the BIC and so it was concluded that there was insufficient evidence to indicate a break point in trend over time.

When sub-grouped by age at death (see Fig. 3), both neonatal and post-neonatal mortality rates experienced downward trends, although the magnitude of improvement was smaller amongst post-neonatal mortality rates and remained relatively static in this sub-group between 2014 and 2018. Similar to IMR, BIC did not indicate break points in trends during the study period in these sub-groups.

Stillbirth rates followed a different trajectory (see Fig. 3). They were relatively static between 2000 and 2009, fluctuating between 5.2–5.6 stillbirths per 1000 births, but began to decline from 2009 onwards, with rates falling to 3.7 stillbirths per 1000 births in 2018. This was supported by break point analysis, as introduction of a break point in 2009 improved the fit of the negative binomial regression model.

### Socio-economic differences in mortality and stillbirth rates

**Fig. 4:**
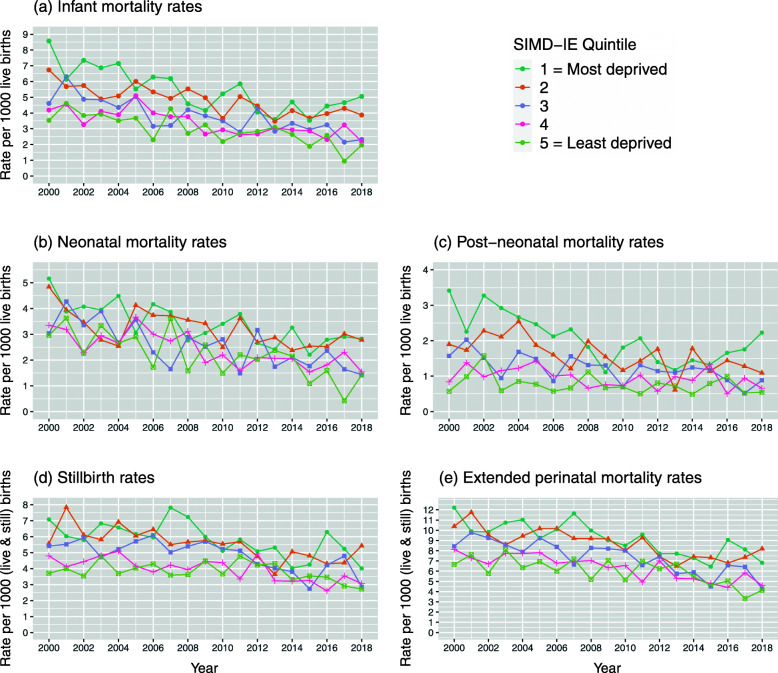
**a**–**e** Trends in mortality and stillbirth rates by SIMD-IE, 2000–2018

**Fig. 5:**
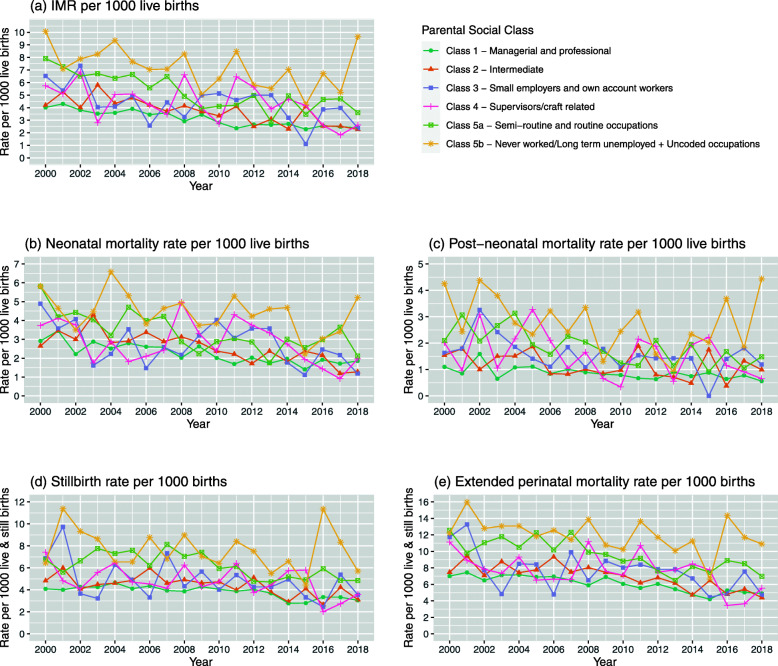
**a**–**e** Trends in mortality and stillbirth rates by NS-SEC, 2000–2018

As presented in Fig. 4, when stratified by SEP, infant mortality and stillbirth rates were consistently higher in the most (Q1) relative to the least deprived SIMD-IE quintile (Q5) between 2000 and 2018. Since 2016, the most deprived quintiles in infant, neonatal and post-neonatal mortality rates have experienced a change in previous downward trajectories, with a rising trend in mortality rates observed. As displayed in Fig. 5, these findings were reproduced when SIMD-IE was replaced with NS-SEC.

In the fully adjusted negative binomial regression models, increasing deprivation was associated with a higher incidence of infant mortality and stillbirth. For example, each unit change in SIMD-IE quintile, from least (Q5) to most (Q1) deprived was accompanied by a 16% increase in the incidence rate of infant mortality (IRR 1.16, 95% CI 1.13–1.19). The greatest inequality was observed amongst post-neonates, with each successive transition from least to most deprived quintile associated with a 25% increase in the incidence rate of post-neonatal mortality. (IRR 1.25, 95% CI 1.20–1.31) (Table 3) The findings were similar when NS-SEC was used to measure SEP: the incidence rate of infant mortality increased by 15% (IRR 1.15, 95% CI 1.13–1.17) with each unit change in NS-SEC class from least disadvantaged (Class 1) to most disadvantaged (Class 5b) and again the magnitude of association was greatest amongst post-neonatal mortality, with an IRR of 1.22 (95% CI 1.19–1.26) (Table 4).

**Table 3:** Predicted incidence rate ratios (IRR) of infant mortality events obtained from negative binomial regression modelling using SIMD-IE

	Model explanatory variables				
	Year	SIMD-IE Quintile	Infant sex	Maternal age	
**Reference category**	*2000*	*5 (least deprived)*	*Female*	*25–34 years*	
**IRR for**	*Unit increase*	*Unit increase*	*Male*	*< 25 years*	*≥ 35 years*
**Infant mortality**	0.97 * (0.96–0.97)	1.16 * (1.13–1.19)	1.22 * (1.15–1.30)	1.24 * (1.16–1.34)	1.16 * (1.07–1.26)
**Neonatal mortality**	0.97 * (0.96–0.97)	1.12 * (1.09–1.15)	1.20 * (1.12–1.30)	1.16 * (1.06–1.27)	1.18 * (1.07–1.30)
**Post-neonatal mortality**	0.97 * (0.96–0.98)	1.25 * (1.20–1.31)	1.26 * (1.13–1.40)	1.42 * (1.26–1.60)	1.12 (0.96–1.30)
**Stillbirths**	0.98 * (0.97–0.98)	1.13 * (1.11–1.15)	0.99 (0.94–1.05)	1.12 * (1.04–1.20)	1.37 * (1.28–1.47)
**Extended perinatal mortality**	0.97 * (0.97–0.98)	1.13 * (1.11–1.14)	1.06 * (1.02–1.11)	1.13 * (1.07–1.20)	1.30 * (1.23–1.38)

* *p*-value < 0.001

**Table 4:** Predicted incidence rate ratios (IRR) of infant mortality events obtained from negative binomial regression modelling using NS-SEC

	Model explanatory variables				
	Year	NS-SEC	Infant sex	Maternal age	
**Reference category**	*2000*	*Class 1 (least disadvantaged)*	*Female*	*25–34 years*	
**IRR for**	*Unit change*	*Unit change*	*Male*	*< 25 years*	*≥ 35 years*
**Infant mortality**	0.97 * (0.96–0.97)	1.15 * (1.13–1.17)	1.22 * (1.15–1.30)	1.10 (1.01–1.18)	1.17 * (1.08–1.27)
**Neonatal mortality**^**a**^	0.97 * (0.96–0.97)	1.12 * (1.10–1.14)	1.21 * (1.12–1.30)		
**Post-neonatal mortality**^**a**^	0.97 * (0.96–0.98)	1.22 * (1.19–1.26)	1.26 * (1.13–1.41)		
**Stillbirths**	0.98 * (0.97–0.98)	1.15 * (1.14–1.18)	1.00 (0.94–1.05)	0.96 (0.90–1.04)	1.40 * (1.30–1.51)
**Extended perinatal mortality**	0.97 * (0.97–0.98)	1.15 * (1.13–1.16)	1.07 * (1.02–1.12)	0.99 (0.93–1.05)	1.32 * (1.25–1.41)

* *p*-value < 0.001

^a^ Note for neonatal mortality BIC for the model with and without adjustment for maternal age was 2651 and 2650, respectively, therefore adjustment for maternal age not included in the model. Similarly, for post-neonatal mortality, BIC for the model with and without adjustment for maternal age was 2137 and 2134, respectively, therefore adjustment for maternal age not included in the model

### Changes in inequality over time

**Fig. 6:**
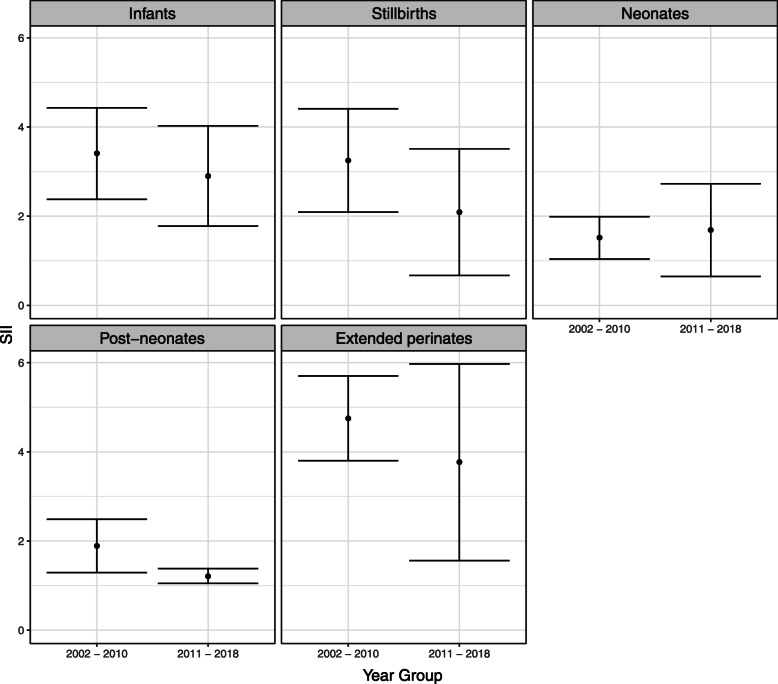
The slope index of inequality (SII) in infant mortality and stillbirth rates by SIMD-IE

**Fig. 7:**
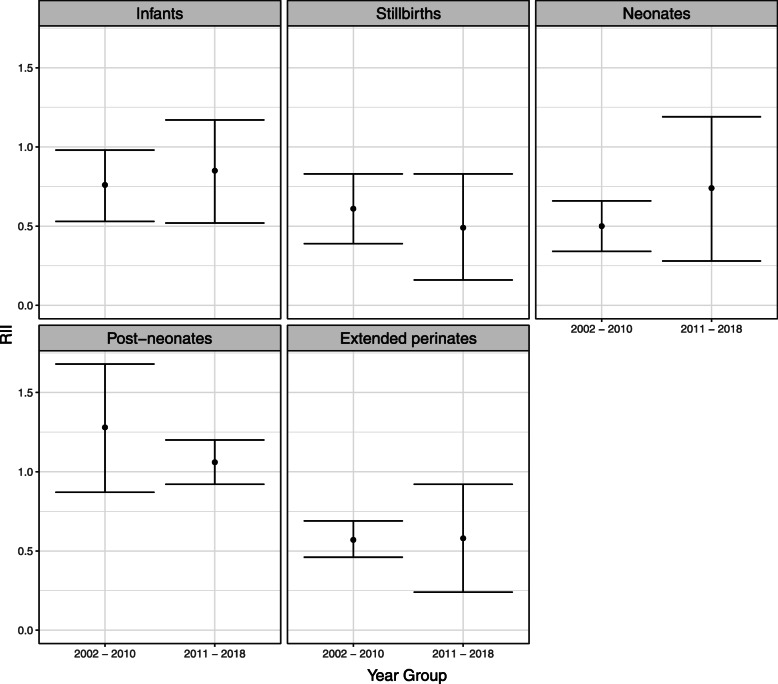
The relative index of inequality (RII) in infant mortality and stillbirth rates by SIMD-IE

**Fig. 8:**
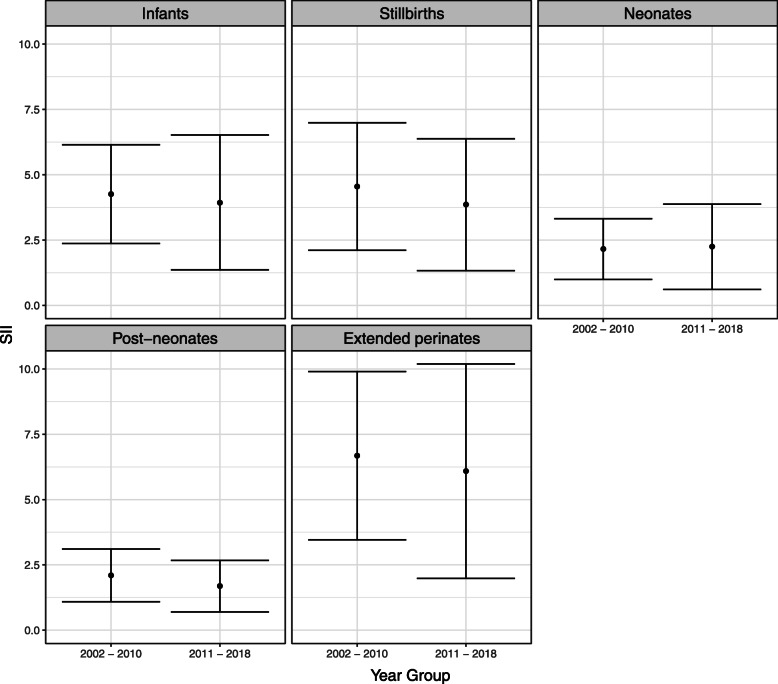
The slope index of inequality (SII) in infant mortality and stillbirth rates by NS-SEC

**Fig. 9:**
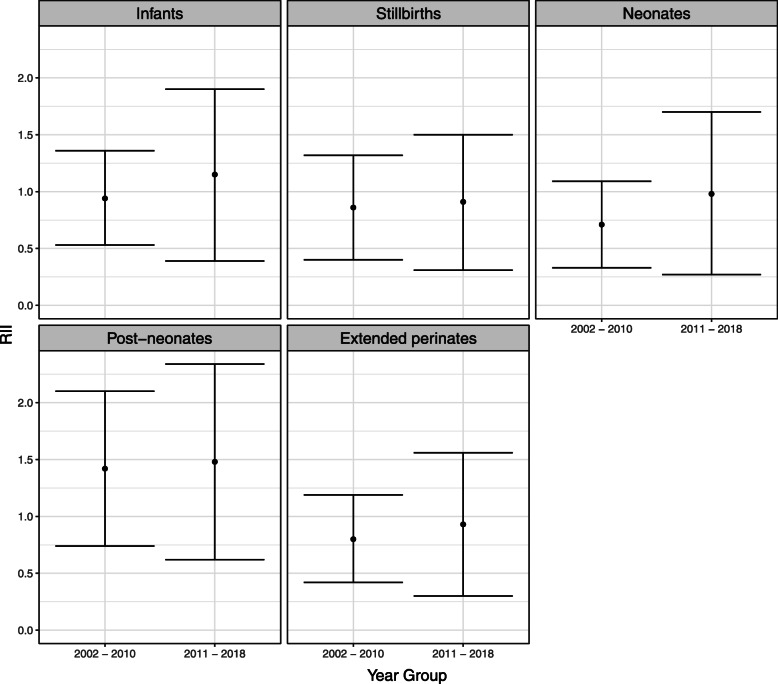
The relative index of inequality (RII) in infant mortality and stillbirth rates by NS-SEC

As displayed in Figs. 6 and 7, respectively, the point estimates of the SII and RII for infant mortality and stillbirth rates by SIMD-IE in the two time periods, 2002–2010 and 2011–2018, were accompanied by wide and overlapping confidence intervals. It was therefore concluded that there was no strong evidence for a change in absolute or relative inequality in infant mortality, neonatal mortality, post-neonatal mortality, extended perinatal mortality or stillbirth rates for the 8-year periods before and after the introduction of the UK Government’s austerity programme. Similar findings were observed in Figs. 8 and 9, respectively, when SIMD-IE was replaced by NS-SEC as the measure of SEP.

### Sensitivity analyses

Findings were similar when analyses were restricted to infants from singleton pregnancies only.

## Discussion

### Main findings of this study

Between 2000 and 2018 Scotland has experienced a downward trend in IMR, with no break point in trend identified. When stratified by SEP, increasing levels of socio-economic disadvantage were associated with higher incidence rates of infant mortality and stillbirth, with the greatest inequality observed amongst post-neonatal mortality. Descriptive analysis showed that whilst infant mortality and stillbirth rates across all SEP groups declined between 2000 and 2015, from 2016 onwards, the most disadvantaged SEP groups have experienced an unusual upward trend in IMR. Finally, when comparing inequalities in infant mortality and stillbirths between the 8-years before and after the introduction of austerity, there was no strong evidence of a change in absolute or relative inequality.

### Strengths and limitations of this study

This study was strengthened by using both an individual and area-based measure of SEP. NS-SEC overcame the risk of ecological fallacy that arises from using SIMD-IE alone, which measures deprived areas rather than individuals, whilst SIMD-IE carried the advantage of being recorded more completely than the NS-SEC. A second strength was the inclusion of stillbirth data; incorporation of stillbirths into the extended perinatal mortality rate overcame the risk of under-estimating IMR due to misclassification bias that arises when live infants who are delivered at or beyond 24^+ 0^ weeks but who die soon after birth are registered as stillbirths instead of live births and infant deaths.

A limitation of the study was that adjustment for variables that may have confounded or modified the relationship between SEP and infant mortality and stillbirths was restricted to the variables that are routinely collected during vital registrations. In the absence of linking vital statistics to maternity records, it was not possible to adjust for additional variables such as ethnicity, maternal body mass index (BMI), congenital anomalies, or gestational age. The latter variable was cited by the literature as the main effect modifier in the relationship between SEP and mortality. As the incidence of premature delivery is higher amongst more disadvantaged groups, adjustment for gestational age in the statistical modelling would likely have attenuated the association between deprivation and mortality. A further limitation was that the absence of information on gestational age meant it was not possible to ensure exclusion of live born infants delivered at < 24^+ 0^ weeks gestation from the study. Infants delivered at < 24^+ 0^ weeks have extremely high mortality rates, and in the UK such events should be registered as live births and infant deaths. There is however evidence that registration practices vary over time and between population groups, and in some instances these events are misclassified as late fetal losses. This variable practice thus creates data instability and so it is usually recommended that to improve validity, infants delivered < 24^+ 0^ should be excluded from analyses [18].

### Explanation of findings and comparison with previous literature

The overall downward trend in IMR in Scotland between 2000 and 2018 is comparable with observed trends across the European Union (EU) between 1994 and 2015. In contrast to England, whilst Scotland did not experience an increase in IMR in recent years, the potential plateauing of trends observed in descriptive analysis from 2015 onwards does mirror findings from the UK overall and in Ireland [19].

Regarding stillbirths, the trends observed in Scottish stillbirth rates were relatively static until 2008, after which time they began to decline. In England, the trend was similar, but the decline began later in 2011. Exploring the UK-wide decline over the past decade, Iliodromiti et al. proposed several explanations, including the smoking ban in public places, national polices on stillbirth reduction and the increased use of third trimester obstetric ultrasound scanning to detect and manage obstetric complications [20].

When stratified by SEP, socio-economic health inequalities have persisted over time and the observation of rising infant, neonatal and post-neonatal mortality rates from 2016 onwards amongst the most disadvantaged SEP groups does mirror English findings [5] and is consistent with international trends in Europe and the USA. For example, between 2004 and 2016 Greece observed disparities in IMR trends between urban and rural infants and cited poorer education and lower income as potential explanations for rural infants experiencing rising IMR [21]. Whilst in California, deleterious effects of the global financial crisis on adverse birth outcomes were more pronounced amongst socioeconomically disadvantaged populations [22]. It is well documented that differences in factors such as maternal behaviours, quality of housing and social support between SEP groups contribute to observed inequalities and that these factors extend beyond individual control and are influenced by the wider socio-economic environments that limit people’s employment opportunities, restrict nutritional options, and cause high levels of stress [23]. The early observation of stabilising and rising trends amongst the most disadvantaged SEP groups may therefore be an early warning that the wider socio-economic environment in Scotland is beginning to disproportionately impact the most vulnerable.

Alternative explanations for these socio-economic trends are that because Scottish rates are based on relatively small numbers of approximately 150–250 infant deaths per year, recent trends may be due to normal annual fluctuations and therefore require longer follow-up. Additionally, observed increases in IMR may be due to the same data artefact that arose in England, whereby the 2014–2017 rise in IMR was attributed to increasing numbers of deaths on day zero of infants born < 24^+ 0^ weeks gestation [4]. However, because increasing IMR in the most deprived groups was observed in both the neonatal and post-neonatal sub-groups, it is unlikely that this explanation fully accounts for the observed patterns because infants born at < 24^+ 0^ weeks gestation would be unlikely to survive into the post-neonatal period.

When socio-economic inequalities were formally assessed using SII and RII, however, the gap in mortality and stillbirth rates between the most and least disadvantaged groups in the 8-years before and after the 2010 introduction of austerity appeared stable. If this is a true finding then whilst it is disappointing that inequalities have not improved, it is a relative achievement that they have not significantly worsened amidst such difficult socio-economic conditions. However, because assessment of the absolute and relative gap in mortality and stillbirth rates was based upon a policy-driven 2010 breakpoint, which was pre-specified in the study protocol, it may be that aggregation of mortality and stillbirth events into a 2011–2018 category masked more recent or subtle changes.

### Implications for public health practice

Action to reduce inequalities in infant mortality and stillbirth rates should draw on the concept of proportionate universalism and direct a higher intensity of resources towards the populations with the greatest need [24]. In Scotland, the most disadvantaged socio-economic groups had the highest rates of mortality and stillbirth events and would therefore warrant a greater proportion of resources. For stillbirths and neonatal deaths, action should focus on reducing the risk of obstetric complications such as growth restriction, congenital anomalies, and prematurity, by creating healthier in-utero environments through interventions to promote maternal health [25]. Reductions in post-neonatal mortality could be achieved by increasing support for caregivers, optimising the environments that infants are raised in and reducing risk factors for adverse events. For example, breast feeding support, improved access to mental health services and financial support for infant-care essentials could improve the environments that infants are raised in [4], whilst individually tailored or community-based interventions to address risk factors such as smoking and sleeping position, could reduce the risk of sudden unexpected deaths in infancy (SUDIs) amongst the most socially vulnerable groups [26].

### Recommendations for future research

As descriptive analysis suggested an uptick in infant, neonatal and post-neonatal mortality rates amongst the most disadvantaged SEP groups from 2016 onwards, further research over a longer period is warranted to explore whether this is a distinct trend rather than a random fluctuation. Updating break-point analysis with a longer time period, and separately for each SEP group would also reveal if 2016 truly did mark a start of change in trends and if different SEP groups are following different trajectories in IMR and stillbirth rates.

Finally, linkage to maternity data that would enable adjustment for gestational age, which would both strengthen the validity of results and better inform policy makers about what population subgroups are experiencing the greatest inequality in health outcomes.

## Conclusions

Socio-economic inequalities in infant mortality and stillbirth rates are persisting in Scotland and recent descriptive trends raise concerns that the most disadvantaged SEP groups may be experiencing worsening trends. As Scotland emerges from the COVID-19 global pandemic and as the UK adjusts to its departure from the EU, its socio-economic environment will be faced with another wave of unavoidable and significant change. As a result, the goal of creating a country where children flourish in their early years will become increasingly difficult and the threat of worsening socio-economic inequalities will grow. Policymakers should therefore use up-to-date data to inform them of the impact that social and fiscal policy can have on child health outcomes and take bold action to protect its children from harm.

## Data Availability

The data that support the findings of this study are available from National Records of Scotland, but restrictions apply to the availability of these data, which were used under license for the current study, and so are not publicly available. Data are however available from the authors upon reasonable request and with permission of National Records of Scotland. All data analysis was undertaken in RStudio version 1.2.5033. The code used to tidy and analyse the data supplied by National Records of Scotland is available on request from the authors.

## Abbreviations

BIC: Bayesian Information Criterion
BMI: Body mass index
EU: European Union
IMR: Infant mortality rate
IRR: Incidence rate ratio
NRS: National Records of Scotland
NS-SEC: National statistics socio-economic classification
RII: Relative index of inequality
SEP: Socio-economic position
SII: Slope index of inequality
SIMD-IE: Scottish Index of Multiple Deprivation income and employment
SUDI: Sudden unexpected death in infancy
UK: United Kingdom
